
JOURNAL INFORMATION
==============================
NLM Title Abbreviation: BMC Neurosci
Journal ID: BMC Neurosci
NLM Journal ID: 100966986

BMC Neuroscience

EISSN: 1471-2202
Publisher: BMC

ARTICLE INFORMATION
==============================
PMCID: PMC3736057
DOI: 10.1186/1471-2202-14-S1-P368
Article ID: 1471-2202-14-S1-P368
Article version: 1
Subjects: Poster Presentation

Abstract withdrawn

Publication date: 2013
Electronic publication date: 2013 Jul 8
Volume: 14
Issue: Suppl 1
Supplement: Abstracts from the Twenty Second Annual Computational Neuroscience Meeting: CNS*2013Gennady S Cymbalyuk and Astrid A PrinzPublication of this supplement has been funded by the Organization for Computational Neurosciences. The Supplement Editors declare that they have no competing interests.
First page: P368
Last page: P368
Copyright: Copyright © 2013 licensee BioMed Central Ltd.
Copyright year: 2013
Copyright holder: licensee BioMed Central Ltd.
License: This is an Open Access article distributed under the terms of the Creative Commons Attribution License (http://creativecommons.org/licenses/by/2.0), which permits unrestricted use, distribution, and reproduction in any medium, provided the original work is properly cited.
License URL: https://creativecommons.org/licenses/by/2.0/

Conference date: 13-18 July 2013
Conference name: Twenty Second Annual Computational Neuroscience Meeting: CNS*2013
Conference location: Paris, France

==============================
*BMC Neuroscience* 2013, **14**(Suppl 1):P368
http://www.biomedcentral.com/1471-2202/14/S1/P368

POSTER PRESENTATION Open Access

### Abstract withdrawn

*From* Twenty Second Annual Computational Neuroscience Meeting: CNS*2013
Paris, France. 13-18 July 2013

Published: 8 July 2013

doi:10.1186/1471-2202-14-S1-P368
**Cite this article as:** : Abstract withdrawn. *BMC Neuroscience* 2013
**14**(Suppl 1):P368.

**Submit your next manuscript to BioMed Central and take full advantage of:**

- **Convenient online submission**
- **Thorough peer review**
- **No space constraints or color figure charges**
- **Immediate publication on acceptance**
- **Inclusion in PubMed, CAS, Scopus and Google Scholar**
- **Research which is freely available for redistribution**

Submit your manuscript at
www.biomedcentral.com/submit

© 2013 licensee BioMed Central Ltd. This is an Open Access article distributed under the terms of the Creative Commons Attribution License (http://creativecommons.org/licenses/by/2.0), which permits unrestricted use, distribution, and reproduction in any medium, provided the original work is properly cited.
